# Will the community nurse continue to function during H1N1 influenza pandemic: a cross-sectional study of Hong Kong community nurses?

**DOI:** 10.1186/1472-6963-10-107

**Published:** 2010-04-30

**Authors:** Eliza LY Wong, Samuel YS Wong, Kenny Kung, Annie WL Cheung, Tiffany T Gao, Sian Griffiths

**Affiliations:** 1 School of Public Health & Primary Care, The Chinese University of Hong Kong, Hong Kong, School of Public Health Building, Prince of Wales Hospital, Shatin, New Territories, Hong Kong, China; 2 Hospital Authority of Hong Kong, 4/F, School of Public Health Building, Prince of Wales Hospital, Shatin, New Territories, Hong Kong, China

## Abstract

**Background:**

Healthcare workers have been identified as one of the high risk groups for being infected with influenza during influenza pandemic. Potential levels of absenteeism among healthcare workers in hospital settings are high. However, there was no study to explore the attitudes of healthcare workers in community setting towards the preparedness to the novel H1N1 influenza pandemic. The aim of this study was to explore the willingness of community nurses in Hong Kong to work during H1N1 influenza pandemic.

**Methods:**

A cross-sectional survey was conducted among all 401 community nurses employed by the Hospital Authority in Hong Kong when the WHO pandemic alert level was 6.

**Results:**

The response rate of this study was 66.6%. 76.9% participants reported being "not willing" (33.3%) or "not sure" (43.6%) to take care of patients during H1N1 influenza pandemic. The self-reported reasons for being unwilling to report to duty during H1N1 influenza pandemic were psychological stress (55.0%) and fear of being infected H1N1 influenza (29.2%). The reported unwillingness to report to duty was marginally significantly associated with the request for further training of using infection control clinical guideline (OR: 0.057; CI: 0.25-1.02). Those who reported unwillingness or not being sure about taking care of the patients during H1N1 influenza pandemic were more depressed (p < 0.001) and found work more emotionally stressful (p < 0.001).

**Conclusions:**

Interventions to provide infection control training and address community nurses' psychological needs might increase their willingness to provide care to patients in the community during H1N1 influenza pandemic. This would help to ensure an effective and appropriate health system response during the H1N1 influenza pandemic.

## Background

The current influenza (H1N1) pandemic has become a public health threat due to its associated morbidity and mortality. Among those at high risk for being infected with influenza (H1N1), healthcare workers have been identified as the priority group whose preparedness is a critical element in the response to the pandemic. In addition to patient care, HCWs are involved in public health education, epidemiological surveillance, quarantine management, fever clinics, staging facility operation, and more [1,2]. Although the role of HCWs is important during emergencies such as a pandemic, not all healthcare workers are ready or prepared to work with infectious patients [3]. For example, during the SARS outbreak, some healthcare workers in Taiwan escaped from hospital or resigned due to perceived shortage of facilities for handling patients with SARS [4]. Since then, a number of studies have been conducted to explore the willingness of HCWs to work during influenza pandemics. These studies suggested that at the time of an epidemic, the potential levels of absenteeism could be as high as 16% in Hong Kong [5], 28% in Germany [6], 33% in Australia [7], 43% in Taiwan [8] and 50% in UK [9]. Potential absenteeism was most likely among nursing and ancillary workers [10] and the major reported reasons were fear for themselves and their families' health and lack of protective equipments [3,11,12].

Healthcare workers who care for patients in their homes are among those at high risk in pandemics. Previous studies in the United States showed that the number of patients who were being cared for at home during a pandemic was nearly 3 times to the number being hospitalized [13]. During the SARS epidemic in Hong Kong, the fall in hospitalizations for those with pre-existing chronic diseases was complemented with an increase for demand for community nursing services at patients' homes [14].

Studies from the United Kingdom found that community HCWs expressed less willingness to work during a pandemic than their hospital counterparts [10]. A similar study in the United States found that the intention to work with quarantine cases among community home health care services was lower (11%) than the willingness of healthcare workers who worked in the hospital (54%) [15]. Another study found that for HCWs working in New York City long-term facilities and outpatient centers, the most important barriers to willingness to work were fear and concern for family and self (31%) [16]. To the best of our knowledge, there is no other study exploring the willingness of, and factors associated with community nurses to work during the H1N1 pandemic. Thus, this study was conducted to explore the willingness of community nurses to continue to work during H1N1 influenza pandemic.

## Methods

### Study design

This cross-sectional survey investigated the willingness of community nurses in Hong Kong to work during H1N1 influenza pandemic. All community nurses in Hong Kong are employed by the Hong Kong Hospital Authority and their responsibility is to provide patient care and education in patients' homes. This programme was introduced in 1967 with the aim of providing continuing nursing care for people who are discharged from hospitals. There are now 401 community nurses working out of 48 centres and stations attached to 14 hospitals over 7 organizational clusters in Hong Kong, each serving a geographical region with a population catchment of approximately 1 million people. Patients are referred to community nursing services by public hospitals and out-patient clinics or through self referral. Community nurses work closely alongside with the community geriatric service nurse in old age homes and the psychiatric outreach team.

### Participants

During 24 to 30 June 2009 when Hong Kong faced community spread of H1N1 and around 50 cases were being reported a day in the community [17], a cross-sectional survey was conducted among all 401 community nurses in Hong Kong to determine their willingness to work during the H1N1 influenza pandemic, when the WHO pandemic alert level was 6. A questionnaire was sent to all community nurses who provided medical care services at patient's home in Hong Kong excluding those working for geriatric teams at old age homes and as part of psychiatric outreach. General Managers of community nursing service centres or stations were contacted by phone to obtain the approval to send the questionnaire to their nursing staff. In total, 401 self-administered anonymous questionnaires were sent to all community nurses via their general managers in the 7 organizational clusters in Hong Kong. To keep responses confidential and anonymous the completed questionnaires were returned to us directly or via the centre/station manager in sealed envelopes.

### Survey design

The content of this questionnaire was based on the conceptual framework suggested by Patel et al [18] and consisted of 6 aspects with 44 questions: (1) clinical services change as a response to pandemic influenza; (2) internal environment changes as a response to pandemic influenza; (3) macro-environmental changes as response to pandemic influenza; (4) professional and public health responsibilities with respect to pandemic influenza; (5) attitude and psychological responses to pandemic influenza; (6) willingness to continue to work during H1N1 influenza pandemic; and (7) demographics and year of education of respondents. This paper only reports the community nurses' willingness to work during H1N1 influenza pandemic and its associated factors. The study was reviewed and approved by the Survey & Behavioural Research Ethics Committee of The Chinese University of Hong Kong.

### Statistical analysis

Descriptive statistics including the characteristics of respondents giving positive or negative responses to their willingness to take care the patients with suspected H1N1 influenza were presented. Responses were dichotomized into those with "willingness to work" and those with "not willingness to work or unsure". Binary logistic regression was used to compute bivariate and multivariate odds ratios (ORs) to evaluate the association of demographic variables (age, working district, post-graduation education, year of nursing registration, years of being community nurses) and other respondent characteristics (use of infection control clinical guideline, received training for infection control clinical guideline, request further training, experiences of taking care SARS or Avian Flu cases, received vaccination in the past 12 months, participation in surveillance) with the willingness. Responses to the stress level using 100 m Visual Analog Scale in 9 items (the higher score, the more stressful) and satisfaction level using 100 m Visual Analog Scale in 2 items (the higher score, the higher the satisfaction) were used to explore the relationship with the respondents to the willingness to work by independent t-test. All data were analyzed using SPSS (version 15.0) and p < 0.05 was considered to be statistically significant.

## Results

### Demographics

A total of 401 self-administered questionnaires were distributed to all community nurses in Hong Kong and 270 completed questionnaires returned within which three questionnaires were uncompleted, within a two week period between 24 Jun, 2009 and 8 July, 2009 (response rate: 66.6%). Of the 267 valid respondents, the majority were females (96.2%) who had been registered nurses for 16.5 years (ranging from 1 year to 36 years) and had worked for an average of 8.8 years as a community nurse (ranging from 2 months to 32 years). The mean age of respondents was 39.1 years and about a third (29.2%) had had the experiences of the taking care of SARS/Avian Flu cases. Two third of them had postgraduate qualifications and one third of them had received vaccination for seasonal influenza in the past 12 months. Nurses from each cluster participated and other demographic details are summarized in Table 1.

**Table 1:** Demographics of nurses in the study

Demographic characteristics	N(%)	Mean(SD)	Range
**Number of community nurses**	267		
**Age**	-	39.13(7.69)	24-60
**Gender**			
*Female*	254(96.2)	-	-
*Male*	10(3.8)	-	-
**Place of Education**			
*Hong Kong*	264(100.0)	-	-
**Year of Registration**	-	16.45(8.45)	1-36
**Postgraduate qualification**	199(76.5)	-	-
*Diploma*	60(34.1)	-	-
*Master*	37(21.0)	-	-
*Special training*	93(52.8)	-	-
*Others*	37(21.0)	-	-
**Work status**			
*Full-Time*	257(98.5)	-	-
*Part-Time*	4(1.5)	-	-
**Years of Working as a community nurse**	-	8.79(6.76)	0.17-32
**District of working**			
*Hong Kong Island*	29(11.2)	-	-
*Kowloon*	120(46.5)	-	-
*New Territories West*	66(25.6)	-	-
*New Territories East*	43(16.7)	-	-

### Willingness to work and characteristics

**Figure 1:**
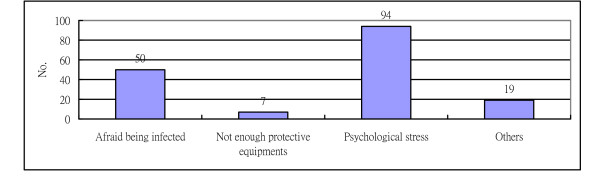
**Reasons of not willingness to work during H1N1 influenza pandemic**.

Overall, one third of participants (36%) reported the demand for community services increased during H1N1 influenza pandemic. 171 participants (76.9%) reported "not willing" (33.3%) or "not sure" (43.6%) about taking care of patients during H1N1 influenza pandemic. The self-reported reasons for being unwilling to take care of patients during H1N1 were psychological stress (55.0%) and fear of being infected (29.2%) (Figure 1). Only one third of participants (33%) had used the infection control clinical guidelines to assist them in taking care of the patients with suspected H1N1 influenza. Almost two thirds of respondents (74.5%) wanted more training and professional education regarding how to deal with H1N1 influenza.

Multivariate analysis of the relationship between professional development and their willingness to take care of patients during H1N1 influenza presented in Table 2 showed that their unwillingness was marginally significantly associated with the request for further training on the use of infection control clinical guideline (OR: 0.51; CI: 0.25-1.02) (Table 2).

**Table 2:** Association between demographics, nurses' behaviours, experiences and their willingness to work during H1N1 influenza pandemic

Variables and variable levels	Bivariate OR (95% CI)	p value	Multivariate OR (95% CI)	p value
***Age***	1.03 (0.99 - 1.07)	0.120	1.08 (0.99-1.19)	0.101
***Postgraduate***				
No	1		1	
Yes	11.50 (0.80 - 2.82)	0.211	11.43 (0.62-3.32)	0.406
***Working as Community Nurse***	1.02 (0.98 - 1.06)	0.409	0.99 (0.93-1.06)	0.822
***Year of Registration***	1.03 (1.00 - 1.06)	0.077	0.96 (0.89-1.03)	0.219
***Use of Guideline***				
No	1		1	
Yes	0.85 (0.50 - 1.47)	0.561	0.76 (0.38-1.49)	0.420
***Further training required***				
No	1		1	
Yes	0.64 (0.35 - 1.17)	0.147	0.51 (0.25-1.02)*	0.057
***SARS or Avian Flu experience***				
No	1		1	
Yes	1.41(0.81-2.45)	0.230	1.18(0.55)-2.53	0.680
***Received vaccination in the past 12 months***				
No	1		1	
Yes	1.03(0.60-1.77)	0.928	0.63(0.30-1.30)	0.209
***Participation in surveillance activity***				
No	1		1	
Yes	1.28(0.70-2.35)	0.427	1.46(0.67-3.15)	0.339

* borderline significant

### Psychological concerns and job satisfaction

Participants who were unwilling or unsure about taking care of patient who was all regarded as "suspected H1N1 cases" generally reported a significantly higher level of psychological stress in various areas including the contraction risk of own and family to H1N1 due to the job, quality of life, activity of living, psychological status and emotional status, in which the details are listed in Table 3. Those who were unwilling or unsure were also significantly more dissatisfied with the influenza case arrangement of the Hospital Authority (unwilling mean score 69.3 mm, willing mean score 74.4 mm, p < 0.049) (Table 3).

**Table 3:** Association between stress levels and their willingness to work during H1N1 influenza pandemic

	Mean	SD	Minimum	Maximum	p-value
***Q28 frightened of dealing with H1N1 influenza**(0, not frightened at all -100, extremely frightening)*					
No or Not Sure	44.18	22.26	0	100	**<0.001**
Yes	21.53	20.09	0	100	
					
***Q29 worried to be infected due to your job**(0, not worrying at all - 100, extremely worrying)*					
No or Not Sure	49.23	22.97	0	100	**<0.001**
Yes	28.92	23.68	0	100	
					
***Q30 worried about infecting your family due to your job **(0, not worrying at all - 100 extremely worrying)*					
No or Not Sure	61.33	25.47	0	100	**<0.001**
Yes	41.99	28.58	0	100	
					
***Q31 confidence that you can protect you and your family from being infected **(0, not confident at all - 100, extremely confident)*					
No or Not Sure	56.07	23.05	0	100	0.091
Yes	61.34	24.52	0	100	
					
***Q32 your family worried about being infected by you due to your job **(0, not worrying at all - 100, extremely worrying)*					
No or Not Sure	50.87	23.99	0	100	**<0.001**
Yes	34.97	27.28	0	100	
					
***Q33 influenza A (H1N1) affected your daily living activities**(0, not affected at all - 100, extremely affected)*					
No or Not Sure	41.06	22.20	0	100	**<0.001**
Yes	27.62	24.94	0	100	
					
***Q34 influenza A (H1N1) affected the quality of your life**(0, not affected at all - 100, extremely affected)*					
No or Not Sure	38.42	22.79	0	100	**<0.001**
Yes	24.25	23.35	0	100	
					
***Q35 feel depressed in the past 2 weeks**(0, not depressed at all - 100 extremely depressed)*					
No or Not Sure	30.33	25.97	0	100	**<0.001**
Yes	17.18	20.27	0	100	
					
***Q36 feel emotionally stressful in the past 2 weeks**(0, not stressful at all - 100, extremely stressful)*					
No or Not Sure	33.16	26.29	0	100	**<0.001**

## Discussion

With the rising morbidity and mortality associated with H1N1 influenza infection, contingency plans are needed since there is an increase in demand on the healthcare workforce in both hospitals and community settings. Absenteeism of HCWs, whether due to fear about work or due to being infected with H1N1 influenza, is one of the major concerns at the time of a pandemic [10]. Studies that aim at improving our understanding of the characteristics and factors that may contribute to HCWs' decision to work or working conditions that may affect their willingness to work during a pandemic are important as they provide a deeper understanding of how to address HCWs needs and keep then engaged in the healthcare system.

Our study found a large proportion of community nurses being unwilling to work during the current influenza pandemic (76.9%) and the figure is much higher than those reported from nurses who work in the hospitals in Hong Kong (16%) [5] and from other countries (28-50%) [6,7,19]. Our findings are, however, consistent with findings from other studies conducted in the United Kingdom [10] which showed that community HCWs including nurses had the lowest reported likelihood of working during an influenza pandemic among all employment categories. The reasons for the higher unwillingness to work during H1N1 pandemic among HCWs in the community are unknown. We may only postulate that the inaccessibility of a protective working environment or facility, for example, isolation rooms with negative pressure may be the concern for HCWs working in patients' homes and further in-depth qualitative studies may be needed to address this issue.

Additional findings from our study showed that community nurses who had inadequate training in infection control were less likely to express a willingness to continue to work. Similar to findings from previous studies conducted in both hospitals and community settings, the major reasons for being unwilling to work during pandemic or outbreak were the risk of infection to self or family and psychological stress [10,16]. The findings demonstrated that stress levels were significantly associated with higher levels of fear of the risk of infection to one's own and/or family health and potential negative impact of H1N1 influenza on nurses' daily living activities and quality of life. Lack of knowledge, ambiguity regarding one's exact tasks, and questionable ability in performing one's role as rick communicator were all significantly associated with a higher perceived personal risk and a two-to ten fold decrease in willingness to report to duty (new suggested one). Special attention should be paid to this group of more junior community nurses who need more training and guidance in dealing with an emerging infectious disease especially in the community setting. Currently infection control training has focused on infection control in the hospital settings, and our findings suggest that regular clinical training should be enhanced for HCWs working in the community in order to increase confidence among all HCWs including nurses in taking care of patients with influenza and reducing the occupational related psychological stress [10]. The findings also showed that the majority of community nurses felt dissatisfied with the arrangements or management of the suspected H1N1 influenza cases (mean score: 71/100) and that unwillingness to work was significantly associated with the reported dissatisfaction. This may suggest that there is a lack of communication between hospital-based management and community HCWs but further studies are needed to explore reasons for dissatisfaction. Experience of SARS highlighted the need for effective communication and HCWs need full access to information as it becomes available [4].

To the best of our knowledge, this is the first study to explore the willingness of community nurses to work during an influenza pandemic and our findings suggest that this is an important issue for policy makers and healthcare organizations especially for those who participate in the provision of community care. Our strength includes an acceptable response rate of 66.6% which is higher than the last survey exploring hospital staff's working attitudes towards influenza pandemic in 2006 in Hong Kong (39%) (CUHK9). The response rate of other western studies covering similar topics was varied ranged from 34% to 79% (H1N1 2, H1N1 3, 3). Therefore, the SAR territory wide representative covers of all nurses working in the community nursing services in Hong Kong.

### Limitation

There were some limitations to this study. First, the marital status and family background of community nurses were not included. Though previous studies showed that HCWs with children were not significantly more likely to be absent, support for child care was reported to be one of the reasons related to the unwillingness to work during influenza pandemic. This may be related to school closure policies rather than hospital management. Nevertheless, this reason was not reported by our participants. Furthermore, we have only reported on the responses to a pandemic among general community nurses employed by the Hospital Authority and, nurses who work in private general practice or with elderly or mentally ill in community centres were not included in our sample. In addition, this was only a cross-sectional study and temporal relationships between unwillingness to work and its associated factors could not be confirmed.

## Conclusions

Our study found that a high proportion of nurses who worked for the community nursing services were unwilling to work with the suspected H1N1 patients. Our study also suggests that community nurses need additional training on infection control in the community setting and are suffering increased psychological stress during the current H1N1 pandemic. The provision of targeted education and improved communication may address these problems and increase their willingness to work during an influenza pandemic.

## Competing interests

The authors declare that they have no competing interests.

## Authors' contributions

All authors were involved in the design of the project. The survey tool was designed by ELYW and SYSW. The data collection and analysis were carried out by AWLC and TGG, with the results interpretation carried by ELYW and SYSW in consultation with SG. The first draft of this article was composed by ELYW and was revised critically by all authors. All authors have approved the final version of the manuscript.

## Pre-publication history

The pre-publication history for this paper can be accessed here:

http://www.biomedcentral.com/1472-6963/10/107/prepub

## References

[B1] Information Services Department H Hong Kong: The Facts - Department of Health 2008 Information Services Department, HKSAR

[B2] Seale H Leask J Po K MacIntyre CR "Will they just pack up and leave?" - attitudes and intended behaviour of hospital health care workers during an influenza pandemic BMC Health Serv Res 2009 9 30 10.1186/1472-6963-9-30 19216792 PMC2661074

[B3] Balicer RD Omer SB Barnett DJ Everly GS Jr Local public health workers' perceptions toward responding to an influenza pandemic BMC Public Health 2006 6 99 10.1186/1471-2458-6-99 16620372 PMC1459127

[B4] Tsai MT Ya-Ti H A resource-based perspective on retention strategies for nurse epidemiologists J Adv Nurs 2008 61 2 188 200 10.1111/j.1365-2648.2007.04463.x 18034819

[B5] Tam DK Lee S Lee SS Impact of SARS on avian influenza preparedness in healthcare workers Infection 2007 35 5 320 325 10.1007/s15010-007-6353-z 17882357 PMC7100841

[B6] Ehrenstein BP Hanses F Salzberger B Influenza pandemic and professional duty: family or patients first? A survey of hospital employees BMC Public Health 2006 6 311 10.1186/1471-2458-6-311 17192198 PMC1764890

[B7] Stuart RL Gillespie EE Hospital pandemic preparedness: health care workers' opinions on working during a pandemic Med J Aust 2007 187 11-12 676 18072917 10.5694/j.1326-5377.2007.tb01472.x

[B8] Tzeng HM Yin CY Nurses' fears and professional obligations concerning possible human-to-human avian flu Nurs Ethics 2006 13 5 455 470 10.1191/0969733006nej893oa 16961111

[B9] Daniels N Duty to treat or right to refuse? Hastings centre report 1991 21 2 36 46 10.2307/3562338 2045281

[B10] Damery S Wilson S Draper H Gratus C Greenfield S Ives J Parry J Petts J Sorell T Will the NHS continue to function in an influenza pandemic? A survey of healthcare workers in the West Midlands, UK BMC Public Health 2009 9 142 10.1186/1471-2458-9-142 19442272 PMC2690584

[B11] Shaw KA Chilcott A Hansen E Winzenberg T The GP's response to pandemic influenza: a qualitative study Fam Pract 2006 23 3 267 272 10.1093/fampra/cml014 16608870

[B12] Yee Wong T Koh GC Cheong SK Sundram M Koh K Chia SE Koh D A cross-sectional study of primary-care physicians in Singapore on their concerns and preparedness for an avian influenza outbreak Ann Acad Med Singapore 2008 37 6 458 464 18618056

[B13] Knebel A Phillips SJ Agency for Healthcare Research and Quality. Home Health Care During an Influenza Pandemic: Issues and Resources 2008 Rockville MD: Agency for Healthcare Research and Quality Publication No 08-0018

[B14] Hospital Authority Hospital Authority Statistical Report 1997 Hong Kong: Hospital Authority, Statistics and Research Section

[B15] Gershon RMR Qureshi AK Stone WP Pogorzelska M Silver A Damsky RM Burdette C Gebbie MK Raveis HV Home Health Care Challenges and Avian Influenza Home Health Care Management Practice 2007 20 1 58 69 10.1177/1084822307305908

[B16] Qureshi K Gershon RR Sherman MF Straub T Gebbie E McCollum M Erwin MJ Morse SS Health care workers' ability and willingness to report to duty during catastrophic disasters J Urban Health 2005 82 3 378 388 16000654 10.1093/jurban/jti086 PMC3456052

[B17] Influenza A(H1N1) InfoDesk http://www3.hku.hk/facmed/h1n1/indexE.php

[B18] Patel MS Phillips CB Pearce C Kljakovic M Dugdale P Glasgow N General practice and pandemic influenza: a framework for planning and comparison of plans in five countries PLoS One 2008 3 5 e2269 10.1371/journal.pone.0002269 18509538 PMC2386973

[B19] NHS Employers and Department of Health Pandemic flu: human resources guidance for the NHS 2008 London: Department of Health

